# Symptom-related clinical phenotypes in adults with obstructive sleep apnea: a scoping review

**DOI:** 10.3389/fmed.2026.1999168

**Published:** 2026-10-29

**Authors:** Xiangke Wang, Xiaoying Yang, Ruilin Li, Xinghao Zhuang, Ting Chen, Geshun Zhou, Hongru Liang

**Affiliations:** 1 School of Nursing, Guangxi University of Chinese Medicine, Nanning, Guangxi, China; 2 School of Basic Medical Sciences, Guangxi University of Chinese Medicine, Nanning, Guangxi, China

**Keywords:** obstructive sleep apnea, positive airway pressure, precision medicine, scoping review, symptom-related clinical phenotypes

## Abstract

**Objective:**

To systematically map the variables and methods used to identify symptom-related clinical phenotypes in adults with obstructive sleep apnea (OSA), summarize the major phenotypes and their associations with treatment and health outcomes, and provide evidence to inform multidimensional clinical assessment and long-term follow-up in OSA.

**Methods:**

This scoping review was reported in accordance with the Preferred Reporting Items for Systematic Reviews and Meta-Analyses extension for Scoping Reviews (PRISMA-ScR). PubMed, Embase, Web of Science, CINAHL, the Cochrane Library, China National Knowledge Infrastructure, Wanfang Data, VIP, and the Chinese Biomedical Literature Database were systematically searched from inception to 27 June 2026. Two reviewers independently screened records and extracted data. Using the symptom classes reported in the original studies as the units of analysis, we synthesized evidence on phenotype-defining variables, identification methods, recurrent symptom patterns across studies, associations with objective disease burden, positive airway pressure (PAP)–related measures, patient-reported outcomes, and long-term clinical outcomes, as well as longitudinal phenotype transitions.

**Results:**

Thirty-six study reports were included. Latent class analysis and cluster analysis were the predominant approaches to phenotype identification. Descriptive cross-study synthesis identified three core phenotypes—daytime sleepiness–predominant, disturbed sleep/insomnia–predominant, and minimally symptomatic—and two extended phenotypes: upper airway/respiratory interruption symptom–predominant and mixed-symptom/high symptom burden. Differences across phenotypes were reported in daytime functioning, psychological status, quality of life, PAP prescription or early use, and symptom improvement following treatment. Symptom burden was not consistently aligned with objective disease burden, and some patients transitioned between symptom phenotypes over time.

**Conclusions:**

Recurrent symptom-related clinical phenotypes were observed across studies of adults with OSA. Symptom phenotypes may serve as a complementary component of multidimensional clinical assessment and should be interpreted alongside objective respiratory and hypoxic burden, comorbidities, and treatment information. They may also inform the assessment of changes in symptoms and functional status during long-term follow-up. Future research should refine phenotype identification, strengthen external validation across populations, and further examine associations of symptom phenotypes with treatment response and long-term outcomes.

## 1 Introduction

Obstructive sleep apnea (OSA) is a common chronic sleep-related breathing disorder in adults, characterized by recurrent upper airway narrowing or collapse during sleep, intermittent hypoxia, and sleep fragmentation. It can impair daytime functioning and is associated with an increased risk of adverse cardiovascular, metabolic, and neurocognitive outcomes (1–3). The apnea–hypopnea index (AHI) remains central to OSA diagnosis and severity assessment. However, it primarily quantifies the frequency of respiratory events and does not adequately capture the multidimensional burden of OSA, including hypoxic exposure, sleep fragmentation, subjective symptoms, and impaired daytime functioning (4). Accordingly, reliance on AHI alone cannot fully account for the substantial clinical heterogeneity of OSA.

As precision medicine research in OSA advances, identifying patient subgroups based on clinical characteristics has become an important approach to understanding disease heterogeneity. Symptom-related clinical phenotypes (hereafter referred to as symptom phenotypes) primarily reflect patients' subjective experiences of disease and associated functional burden. They provide a patient-centered perspective that can be assessed through routine clinical evaluation and patient-reported outcome measures. Previous studies using latent class analysis (LCA), latent profile analysis, cluster analysis, and empirically defined classifications have identified several symptom phenotypes, including daytime sleepiness–predominant, disturbed sleep/insomnia–predominant, and minimally symptomatic phenotypes, across countries and in both clinical and community populations (5–12). Rather than replacing AHI or other objective physiological measures, symptom phenotypes may provide complementary information for a more comprehensive assessment of patients' symptom and functional burden.

Previous reviews have highlighted the heterogeneity of OSA phenotypes and their potential implications for precision medicine (13), while more recent work has summarized the recurrence and clinical associations of symptom-based subtypes across diverse populations (14). However, substantial heterogeneity in phenotype-defining variables, assessment tools, analytical methods, and class labels, together with the use of overlapping cohorts in some studies (15–19), complicates the synthesis of study-defined symptom classes and the assessment of recurrent symptom patterns across independent populations. Moreover, differences in symptom phenotypes do not consistently correspond to differences in AHI or hypoxic burden. Reported associations with positive airway pressure (PAP) prescription or use, symptom changes following treatment, patient-reported outcomes, and selected clinical outcomes are also inconsistent. Limited longitudinal evidence further suggests that some patients transition between phenotypes over time (9, 19, 20). Symptom phenotypes therefore cannot yet be considered established tools for treatment selection or prognostic prediction, and their clinical utility in OSA risk stratification and long-term management remains to be clarified.

Against this background, we conducted a scoping review to systematically map the variables, assessment tools, and methods used to identify symptom phenotypes in adults with OSA, examine how original symptom classes correspond across studies, and assess recurrent symptom patterns across independent study populations while accounting for overlapping cohorts. We also summarized evidence on associations with objective disease burden, treatment implementation and response, patient-reported outcomes, and long-term clinical outcomes, as well as longitudinal phenotype transitions. Through this synthesis, we aimed to examine the potential role of symptom phenotypes as a complementary dimension to conventional objective physiological assessment and to explore the current evidence, limitations, and future research directions regarding their role in multidimensional clinical assessment and long-term follow-up in OSA.

## 2 Materials and methods

### 2.1 Study design

This scoping review was conducted in accordance with the Joanna Briggs Institute (JBI) methodological framework for scoping reviews (21) and reported following the Preferred Reporting Items for Systematic Reviews and Meta-Analyses extension for Scoping Reviews (PRISMA-ScR).

### 2.2 Research questions

In line with its objectives, this review addressed the following research questions: (1) What phenotype-defining variables, assessment tools, and identification methods have been used in studies of symptom phenotypes in adults with OSA? (2) Which symptom phenotypes have been identified across studies, and which symptom patterns recur across independent study populations? (3) What associations have been reported between symptom phenotypes and objective disease burden, treatment implementation and response, patient-reported outcomes, and long-term clinical outcomes? (4) What patterns of symptom phenotype stability and transition have been observed during longitudinal follow-up, and what are the potential implications of this evidence for multidimensional clinical assessment and long-term follow-up in OSA?

### 2.3 Eligibility criteria

The inclusion and exclusion criteria were developed using the Population–Concept–Context (PCC) framework recommended for scoping reviews (21–23).

Inclusion criteria: (1) Population: Adults aged ≥18 years with OSA were eligible; OSA was required to be established according to diagnostic criteria explicitly reported in the original study, using polysomnography (PSG), home sleep apnea testing (HSAT), respiratory polygraphy (PG), or other clearly specified clinical diagnostic criteria for adult OSA. (2) Concept: The review focused on symptom-related clinical phenotypes of OSA, referred to herein as symptom phenotypes. Eligible studies were required to incorporate at least one symptom or symptom-related patient-reported measure directly into phenotype construction, class identification, or the definition of prespecified classification rules, and to report the symptom composition or characteristics of the resulting classes. (3) Context: Eligible settings included sleep centers, hospital outpatient and inpatient services, community and home settings, and other clinical or health management settings relevant to adults with OSA.

Exclusion criteria: (1) Studies primarily involving central sleep apnea, obesity hypoventilation syndrome, or other sleep disorders were excluded if data for participants with OSA could not be extracted separately. (2) Studies that identified individuals at high risk of OSA solely using screening instruments, such as the STOP-Bang or Berlin questionnaire, without an established OSA diagnosis were also excluded. (3) Studies focusing exclusively on pathophysiological endotypes, such as loop gain, arousal threshold, pharyngeal muscle responsiveness, or upper airway collapsibility, were excluded. (4) Reviews, conference abstracts, case reports, reports for which the full text was unavailable, and publications in languages other than Chinese or English were excluded.

### 2.4 Literature search and study selection

PubMed, Embase, Web of Science, CINAHL, the Cochrane Library, China National Knowledge Infrastructure, Wanfang Data, VIP, and the Chinese Biomedical Literature Database were systematically searched from inception to 27 June 2026, using a combination of controlled vocabulary and free-text terms. The complete search strategies for all databases are provided in Supplementary Table S1. Retrieved records were imported into Zotero for deduplication. Two reviewers independently screened titles and abstracts against the eligibility criteria and subsequently assessed the full texts of potentially eligible reports to determine final inclusion. Disagreements were resolved through discussion with a third reviewer, who adjudicated when necessary. Data extracted from the included reports comprised authors, publication year, country, study design and setting, sample size, OSA diagnostic criteria and severity, phenotype identification methods, phenotype-defining variables, the number and original class labels, PAP prescription and use, symptom changes following treatment, cardiovascular, cerebrovascular, and metabolic outcomes, and phenotype stability and transitions.

### 2.5 Data analysis and phenotype synthesis

The characteristics of the included studies were summarized descriptively. Original classes reported in each study served as the units of analysis for cross-study categorization and descriptive synthesis according to prespecified rules (24–26). We use “class” to refer to patient groups identified within the original studies using latent class analysis, cluster analysis, or related methods, whereas “symptom phenotype” is used for cross-study synthesis and clinical interpretation. Two reviewers independently extracted the original class labels, phenotype-defining variables, and symptom characteristics. When original class labels were explicit and consistent with the reported class characteristics, the original authors' definitions were prioritized. When labels were ambiguous or reflected multiple dimensions, the predominant symptom domain was determined using class-specific item-response probabilities, within-class means or standardized scores, cluster centroids, and the authors' descriptions. When measurement instruments or scoring methods differed across studies, mapping decisions were based on the relative distribution of symptoms across classes within each study.

Classes sharing the same predominant symptom domain but differing only in severity were mapped to the same overarching phenotype. Classes with elevated burden in at least two symptom domains and no clearly predominant domain were mapped to the mixed-symptom/high symptom burden phenotype. Classes with low overall symptom burden and no prominent symptom domain were mapped to the minimally symptomatic phenotype. Classes driven solely by demographic characteristics, comorbidities, anatomical features, or sleep-study measures, without a clear symptom pattern, were excluded from the symptom phenotype synthesis. Different classes within the same study could be mapped to different synthesized phenotypes. Two reviewers independently performed cross-study mapping, with disagreements resolved through discussion or adjudication by a third reviewer.

Synthesized phenotypes were classified as core or extended according to their recurrence across independent study populations and the consistency of their symptom composition. Recurrent phenotypes with relatively consistent symptom patterns were classified as core phenotypes, whereas other recurrent phenotypes with lower consistency were classified as extended phenotypes. This classification was used only for descriptive cross-study synthesis in this review, was not based on a prespecified numerical threshold for the number of contributing studies, and should not be interpreted as having undergone formal external validation or as representing an established standardized clinical classification system. For each synthesized phenotype, both the number of study reports and the number of independent study populations were reported. Reports derived from the same or partially overlapping cohorts were conservatively counted as representing a single independent study population. The study-level mapping of original classes to the synthesized phenotypes, together with the rationale for each mapping decision, is presented in Supplementary Table S2.

## 3 Results

### 3.1 Study selection and characteristics of the included studies

The search identified 1,930 records, of which 36 study reports were included (5–12, 15–20, 27–48). The study selection process is presented in Figure 1. The included studies were published between 2014 and 2026 and predominantly recruited participants from sleep centers or specialist outpatient clinics. Sample sizes ranged from 100 to 23,418 participants. Twenty-two reports described cross-sectional studies or cross-sectional analyses of cohort baseline data, and 14 described prospective or retrospective longitudinal cohort studies. Men accounted for more than 50% of the reported sample in 33 studies; two studies included only women, and one reported a male proportion of 46.2%. Overall, the included samples were predominantly male. The characteristics of the included studies are summarized in Table 1.

**Figure 1:**
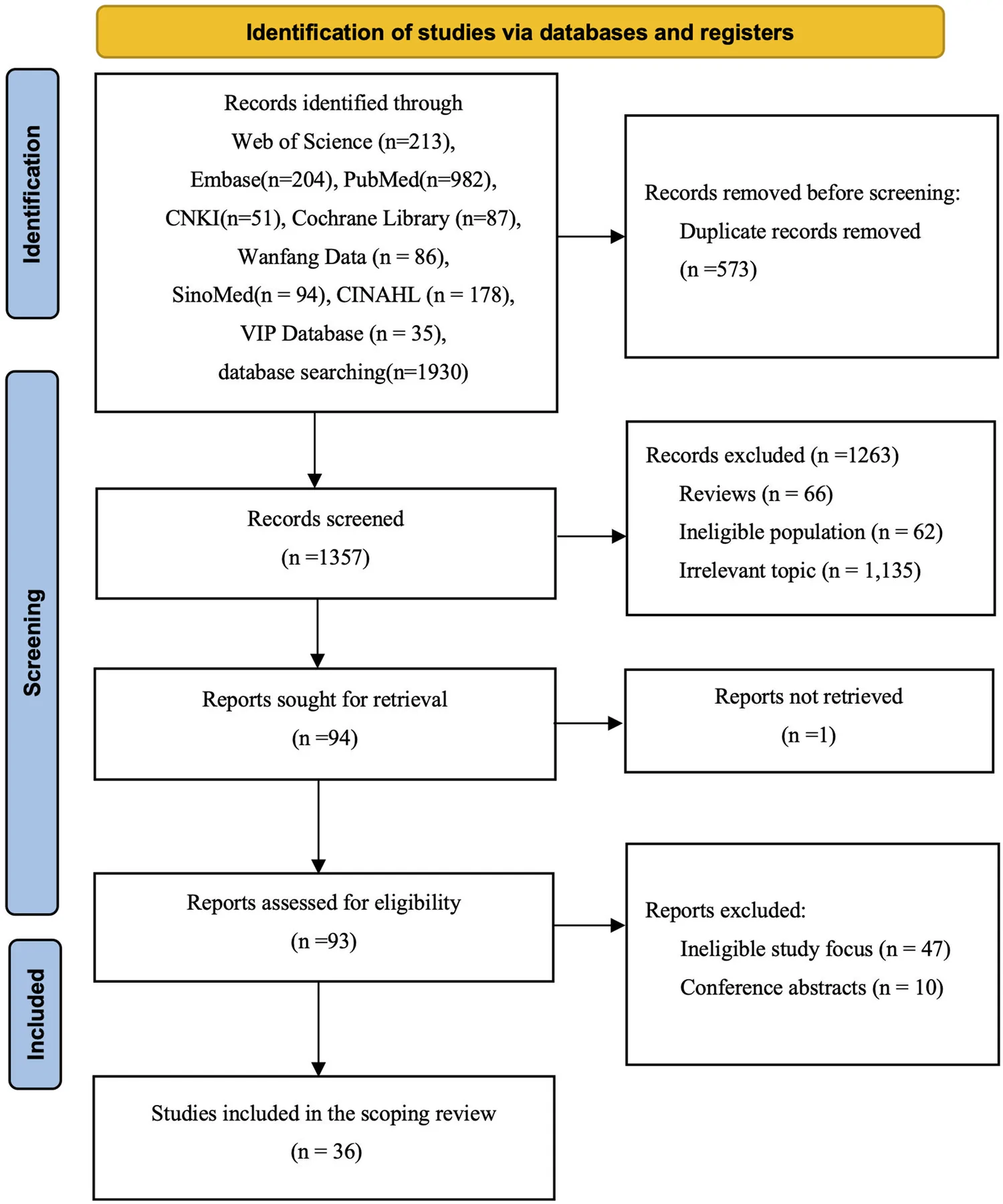
PRISMA flow diagram of study selection.

**Table 1:** Characteristics of the included studies (*n* = 36).

Study	Country or region	Study design and setting	Sample size, *n*	Age and sex	OSA diagnosis and severity	Phenotype variables and instruments	Identification method	Number and original class labels	Main outcomes
Li (41)	China	Cross-sectional study; multiple sleep centers	400	44.9 ± 11.9 years; male 90.5%	PSG; AHI>5 events/h; median AHI 27.1 events/h	Seven PSQI components	LPA	4 classes: Minimal sleep disturbance; Mild sleep disturbance; Moderate sleep disturbance; Severe sleep disturbance	PHQ-9, GAD-7, ESS, CPSS and SSRS
Keenan (7)	International multicenter study	Cross-sectional study; multiple sleep centers (SAGIC)	972	51.9 ± 12.9 years; male 70.3%	PSG/HSAT; AHI≥15 events/h; AHI 42.9 ± 26.8 events/h	①–⑦, ⑨–⑬, ⑮; ESS, BNSQ; B	LCA	5 classes: Disturbed sleep; Minimal symptoms; Upper airway symptoms with sleepiness; Upper airway symptoms dominant; Sleepiness dominant	Comparisons across phenotypes
Pien et al. (9)	Iceland	Prospective cohort study; sleep center (ISAC)	706	54.7 ± 10.3 years; male 80.6%	HSAT; AHI≥15 events/h; AHI 45.9 ± 19.9 events/h	①–⑦, ⑨–⑬, ⑮; ESS, BNSQ, SF-12; B	LCA	3 classes: Disturbed sleep; Minimally symptomatic; Sleepy	PAP adherence, symptoms, blood pressure, SF-12 and BMI
Shi (44)	China	Cross-sectional study; community and multiple sleep centers	2,072	42.8 years; male 73.8%	PSG/HSAT; AHI≥5 events/h; AHI 32.4 events/h	⑤–⑦; ISI; C, D	Two-step clustering	3 classes: Moderate OSA and moderate insomnia; Moderate OSA and severe insomnia; Severe OSA and moderate insomnia	HOMA-IR, lipids, CRP, PHQ-9, GAD-7 and PVT
Bailly (15)	Europe (17 countries)	Cross-sectional analysis of cohort baseline data; multiple sleep centers (ESADA)	23,139	median 55.0 years; male 73.0%	PSG/PG; AHI>5 events/h; Across phenotypes: AHI 23–61 events/h	①; ESS; A–D	LCA	8 classes: C1: Young, obese and sleepy; C2: Older obese men; C3: Obesity with comorbidities; C4: Older and overweight; C5: Pulmonary disease; C6: Young overweight men; C7: Overweight women; C8: Older obese women	Prescription of PAP, MAD, surgery or weight loss interventions
González (6)	United States	Cross-sectional analysis of cohort baseline data; community (HCHS/SOL)	1,605	52.4 ± 13.9 years; male 66.0%	HSAT; REI_3_%≥15 events/h; REI 33.8 ± 22.5 events/h	①–⑦, ⑨–⑬, ⑮; ESS; B	LCA	3 classes: Minimally symptomatic; Excessive sleepiness; Disturbed sleep	Symptoms, SF-12, and cardiovascular, cerebrovascular and pulmonary comorbidities
Tempaku (10)	Brazil	Prospective population-based cohort study; community (EPISONO)	141	51.8 ± 11.9 years; male 56.7%	PSG; AHI≥15 events/h; AHI 30.1 ± 16.8 events/h	①②③⑤⑥⑦⑨⑪⑬⑮; ESS; B	LCA	3 classes: Sleepy; Minimally symptomatic; Disturbed sleep	8-year follow-up: metabolic syndrome, hypertension, diabetes, dyslipidemia and phenotype transitions
Romero-Peralta (43)	Spain	Retrospective cross-sectional study; sleep center (apnea-HUGU)	1,655	52.0 ± 13.0 years; male 64.9%	PSG/HSAT; AHI>5 events/h; AHI 32.2 ± 24.7 events/h	①③④⑥⑨⑩; ESS; A–C	Ward hierarchical clustering	4 classes: Young men without comorbidities, with moderate OSA and ear, nose and throat abnormalities; Symptomatic middle-aged obese men with very severe OSA and comorbidities; Nonobese women with mood disorders; Symptomatic obese men with cardiovascular disease and severe OSA	CPAP, surgery, DISE, and management of cardiovascular disease and mood disorders
Pataka (16)	Europe (17 countries)	Cross-sectional analysis of cohort baseline data; multiple sleep centers (ESADA)	7,288	median 58.0 years; male 0% (100% female)	PSG/PG; AHI≥5 events/h; median AHI 23.9 events/h	①; ESS; A–C	LCA	4 classes: Cluster 1: Women with ischemic heart disease; Cluster 2: Older women with comorbidities; Cluster 3: Sleepy obese women; Cluster 4: Women with mild OSA and a low comorbidity burden	Prescription of PAP, oral appliances, surgery or weight loss interventions
Saaresranta (17)	Europe (17 countries)	Prospective cohort study; multiple sleep centers (ESADA)	6,555	51.5–54.6 years; male 75.4%	PSG/PG; AHI≥5 events/h; Across phenotypes: AHI 24.0–37.4 events/h	①⑤⑧; ESS; insomnia diagnosis; hypnotic use	Empirical classification	4 classes: EDS (daytime+/nighttime−); Non-EDS/non-insomnia (daytime−/nighttime−); EDS/insomnia (daytime+/nighttime+); Insomnia (daytime−/nighttime+)	CPAP use duration and adherence
Fontanilles Arbones (37)	Spain	Retrospective cross-sectional study; sleep center	1,886	median 59 years; male 0% (100% female)	PSG/PG; AHI>5 events/h; median AHI 23.7 events/h	①③④⑩⑪⑫⑭⑰; ESS; A–C	Hierarchical clustering (Gower distance)	4 classes: Middle-aged women with few symptoms and no cardiovascular risk; Older women with few symptoms, cardiovascular disease and severe OSA; Middle-aged women with classic symptoms and cardiovascular risk; Middle-aged women with mood disorders and non-restorative sleep	Comparisons across phenotypes
Teunissen (45)	Netherlands	Cross-sectional study; sleep center	151	median 57.2 years; male 76.8%	PSG; AHI≥15 events/h; AHI 27.7 events/h	①③⑨⑰⑱⑳; PRAQ; B, C	Two-step clustering	5 classes: No comorbidity; Hypertension; High symptom severity; Low symptom severity; Unclassified (atrial fibrillation-related)	CPAP indications, symptom burden and prediction of treatment response
Allen (5)	Canada	Retrospective cohort study; sleep center	1,292	49.5 ± 11.8 years; male 66.1%	PSG; AHI≥5 events/h; AHI 22.3 events/h	①②③⑤⑥⑨⑮; ESS	LCA	4 classes: Excessively sleepy with disturbed sleep; Moderate sleepiness with disturbed sleep; Disturbed sleep without excessive sleepiness; Minimally symptomatic	First cardiovascular or cerebrovascular events; PAP adherence
Alshumrani (20)	Saudi Arabia	Retrospective cross-sectional study; sleep center	650	49.0 ± 16.0 years; male 57.0%	PSG; AHI≥5 events/h; AHI 29.0 ± 24.0 events/h	①⑤⑥; classification rules based on the chief complaint	Rule-based classification (ISAC framework)	3 classes: Sleepy group; Insomniac group; Minimally symptomatic group	CPAP adherence
Henríquez-Beltrán (39)	Chile	Cross-sectional analysis of cohort baseline data; sleep center (SantOSA)	1,167	median 53–55 years; male 80.8%	HSAT; RDI≥5 events/h; severity summary not reported	①–⑤, ⑨⑩⑮; ESS/SHHS questionnaire; B	LCA	3 classes: Non-sleepy; Disturbed sleep; Excessive daytime sleepiness	Distributions of obesity, comorbidities and ESS scores
Bailly (36)	France	Cross-sectional study; multiple sleep centers (OSFP registry)	18,263	median 59 years; male 72.8%	AHI>15 events/h; median AHI 35 events/h	①③⑧⑨⑫⑭⑱; ESS, Pichot scale; A, B	MCA followed by agglomerative hierarchical clustering	6 classes: The young symptomatic; The older obese; The MD old obese; The young snorers; The drowsy obese; The MD obese symptomatic	Near-miss traffic accidents, hypertension, and potential CPAP adherence and response
Kim (8)	South Korea	Cross-sectional analysis of cohort baseline data; community (KoGES)	422	61.5 ± 7.2 years; male 68.5%	HSAT; AHI≥15 events/h; AHI 25.6 ± 10.8 events/h	①–⑦, ⑨–⑬, ⑮; ESS; B	LCA	3 classes: Disturbed sleep; Minimally symptomatic; Sleepy	Distributions of insomnia, sleepiness, hypertension and diabetes
Lammintausta (18)	Europe (17 countries)	Prospective cohort study; multiple sleep centers (ESADA)	23,418	30.0–79.0 years; male 72.9%	PSG/PG; AHI≥5 events/h; Across phenotypes: AHI 34–36 events/h	①⑤⑧; ESS; insomnia diagnosis; hypnotic use	Empirical classification	4 classes: EDS without sleep complaints; Non-EDS, non-insomnia; EDS-insomnia; Insomnia phenotype	PAP use and changes in ESS and CGI-S scores
Ida (40)	Japan	Retrospective cross-sectional study; sleep center	712	53.9 ± 1.0 years; male 83.3%	PSG/HSAT; AHI>15 events/h; AHI 47.4 ± 1.9 events/h	①; ESS; A–C	Ward hierarchical clustering	5 classes: Classic OSA; Older adult and severe OSA; Milder classic OSA; Excessive sleepiness; Nonobese and minimally symptomatic	Total sleep time, sleep efficiency and sleep stages
Gao (38)	China	Cross-sectional study; sleep center	1,483	50.1 ± 13.8 years; male 75.6%	HSAT; AHI≥5 events/h; AHI 28.8 ± 20.8 events/h	①④⑤⑥⑦⑨ ⑩⑪⑬⑰; ESS	LCA	4 classes: Excessively sleepy (ES); Moderately sleepy with disturbed sleep (ModSwDS); Moderately sleepy (ModS); Minimally symptomatic (MinS)	Diabetes, dyslipidemia, hypertension, coronary heart disease and heart failure
Morris (42)	United States	Cross-sectional analysis of cohort baseline data; community (SHHS)	1,718	64.9 ± 10.9 years; male 53.6%	PSG; AHI 5.0–14.9 events/h; AHI 9.1 ± 2.9 events/h	①–⑦, ⑨⑪⑬⑯; ESS	LCA	4 classes: Minimally symptomatic; Disturbed sleep; Moderately sleepy; Excessively sleepy	Sex-specific phenotype distributions
Van Overstraeten (46)	Belgium	Retrospective cohort study; sleep center	567	54.0 ± 13.0 years; male 67.0%	PSG; AHI≥15 events/h; AHI 42.69 ± 26.29 events/h	①②⑪⑰; ESS; A–D	FAMD followed by clustering	5 classes: Less severe OSAS; Healthier severe OSAS; Poorly sleeping OSAS patients with cardiometabolic comorbidities; Younger obese men with sleepiness at the wheel; Sleepy obese men with very severe desaturating OSAS and cardiometabolic comorbidities	6-month follow-up: CPAP adherence and daily use duration
Ye (12)	Iceland	Cross-sectional analysis of cohort baseline data; sleep center (ISAC)	822	54.5 ± 10.6 years; male 81.0%	HSAT; AHI≥15 events/h; AHI 44.9 ± 20.7 events/h	①–⑦, ⑨–⑬, ⑮⑯; ESS, BNSQ; B	LCA	3 classes: Disturbed sleep; Minimally symptomatic; Excessive daytime sleepiness	SF-12, hypnotic use and comorbidity burden
Yassen (19)	Europe (21 countries)	Prospective cohort study; multiple sleep centers (ESADA)	20,164	54.1 ± 12.2 years; male 73.0%	PSG/PG; AHI≥5 events/h; median AHI 27.3 events/h	⑤⑧; A, B	PAM clustering	7 classes: Obese men with cardiometabolic comorbidities; Obese men with cardiovascular comorbidities; Young, healthy symptomatic men; Multimorbid obese postmenopausal women; Older multimorbid obese men; Symptomatic men with psychiatric disorders; Healthy symptomatic women	PAP adherence and changes in AHI, ESS and BMI
Yi (11)	International multicenter study	Cross-sectional study; multiple sleep centers (SAGIC)	1,863	Chinese group: median 46.6 years, male 82.1%; White group: median 55.5 years, male 69.8%	PSG/HSAT; AHI≥15 events/h; Chinese group: 45.8 events/h, White group: 38.6 events/h	①–⑦, ⑨–⑬, ⑮; ESS; B	LCA	5 classes: Minimally symptomatic; Disturbed sleep; Severely sleepy; Moderately sleepy; Upper airway symptoms dominant	Distributions of symptoms, ESS scores and comorbidities
Guo (32)	China	Retrospective cohort study; sleep center	2,592	43.7 ± 12.7 years; male 90.2%	PSG; AHI≥5 events/h; AHI 43.4 ± 26.7 events/h	①③⑤⑦⑨–⑫⑭⑮⑰; ESS, MMSE, MoCA; B	LCA	4 classes: Nocturnal respiratory interruption group; Daytime sleepiness group; Insomnia and memory impairment group; Minimally symptomatic group	Differences in age, body habitus and PSG parameters
Xia (35)	China	Cross-sectional study; multiple sleep centers	1,331	39–40 years; male 79.0%–84.0%	PSG; AHI≥5 events/h; median AHI 39.0–43.6 events/h	①⑤–⑦⑱; ESS, ISI, SAS, SDS; C, D	K-means clustering	4 classes: Typical sleepiness; Asymptomatic hypoxemia; High symptom burden with mild physiological abnormalities; Symptom perception-dominant	Treatment adherence, illness perceptions, CPAP response and psychological needs
Tang (34)	China	Retrospective cross-sectional study; sleep center	1,008	51.2 ± 11.4 years; male 83.83%	PSG; AHI>15 events/h; AHI 42.2 ± 14.9 events/h	①③⑤–⑦⑩⑪⑬⑭⑱; ESS; B	K-means clustering	3 classes: Daytime sleepiness; Nocturnal insomnia; Minimally symptomatic	Diagnostic delay
Qiu (33)	China	Retrospective cohort study; sleep center	491	49.9 ± 11.0 years; male 88.4%	PSG; AHI≥5 events/h; Across phenotypes: AHI 39.8–56.8 events/h	①③⑤–⑦⑨–⑲; ESS	LCA	4 classes: Respiratory interruptions with daytime sleepiness; Minimally symptomatic; Severely impaired daytime functioning; Comorbid insomnia	Changes in sleep architecture and hypoxemia measures during the first CPAP titration night
Zhang (48)	China	Prospective cohort study; sleep center	291	44.1 ± 11.6 years; male 82.5%	PSG; AHI≥15 events/h; AHI 44.3 ± 25.4 events/h	①; ESS; A, C–E	K-means clustering	3 classes: Normal weight, non-sleepy, moderate OSA; Obese, non-sleepy, severe OSA; Obese, sleepy, very severe OSA with craniofacial restriction	PAP pressure and use, residual AHI, mask leak and comorbidities
Dai (31)	China	Cross-sectional study; sleep center	154	44.7 ± 10.9 years; male 87.7%	PSG; AHI≥5 events/h; severity summary not reported	①③–⑦⑨–⑰⑲; ESS, PSQI; B	Q-mode hierarchical clustering	3 classes: Disturbed sleep group; Daytime sleepiness group; Minimally symptomatic group	Sleep efficiency, arousal index and low arousal threshold
Trzepizur (28)	France	Longitudinal cohort study; multicenter clinical sleep cohort	5,358	median 60.0 (51.0–69.0) years; male 63.7%	PSG/HSAT; AHI>5 events/h; median AHI 27 (14–42) events/h	①④⑤–⑦⑨⑩⑫⑱; ESS	LCA	4 classes: Minimally symptomatic; Disturbed sleep; Excessively sleepy; Moderately sleepy	Major adverse cardiovascular events and all-cause mortality
Morris (29)	United States	Prospective cohort study; community (SHHS)	2,619	62.4 ± 10.5 years; male 46.2%	PSG; AHI≥5 events/h; 9.3 ± 12.0 events/h	①–⑦, ⑨, ⑪, ⑬, ⑯; ESS	LCA	4 classes: Minimally symptomatic; Disturbed sleep; Moderately sleepy; Excessively sleepy	5-year phenotype stability and transitions; associations of age, sex, BMI and AHI with transitions
Mazzotti (27)	United States	Prospective cohort study; community (SHHS)	1,207	66.0 ± 10.5 years; male 67.3%	PSG; AHI>15 events/h; AHI 30.7 ± 16.9 events/h	①–⑦⑨⑪⑬⑯; ESS	LCA	4 classes: Minimally symptomatic; Disturbed sleep; Moderately sleepy; Excessively sleepy	Cardiovascular disease during follow-up, including coronary heart disease, heart failure, stroke and cardiovascular death
Labarca (30)	Chile	Prospective cohort study; sleep center (SantOSA)	788	approximately 55.4 years; male 78.7%	HSAT; REI≥15 events/h; severity summary not reported	①⑨⑩; ESS; A, B, E	LCA	4 classes: Symptomatic men with major comorbidities; Symptomatic women with comorbidities; Asymptomatic men with cardiovascular comorbidities; Young symptomatic men without major cardiovascular comorbidities	Cardiovascular mortality and mortality risk across clinical phenotypes
Venkatnarayan (47)	India	Cross-sectional study; sleep center	100	49.5 ± 13.3 years; male 66.0%	PSG; AHI>5 events/h; AHI 36.8 ± 30.1 events/h	①⑤–⑦⑨⑩; ESS; A–C, E	Two-step clustering	3 classes: Older obese patients with severe OSA and a high comorbidity burden; Younger patients with less obesity and positional OSA; Middle-aged obese patients with prominent symptoms	Comparisons across phenotypes

Phenotype construction variables are coded to reduce repetition. These codes were assigned for this review and do not correspond to variable numbers in the original studies.

Symptom codes: ① daytime sleepiness/drowsiness; ② involuntary sleep, dozing in specific situations, napping or sleepiness while driving; ③ fatigue/lack of energy; ④ non-restorative sleep/feeling unrefreshed on awakening; ⑤ difficulty initiating sleep; ⑥ difficulty maintaining sleep/frequent nocturnal awakenings; ⑦ early morning awakening; ⑧ subjective sleep duration, sleep quality or sleep latency; ⑨ snoring; ⑩ witnessed apneas/nocturnal breathing pauses; ⑪ nocturnal gasping, choking or dyspnea; ⑫ morning headache, dizziness or dry mouth; ⑬ nocturnal sweating; ⑭ nocturia/enuresis; ⑮ restless sleep, nightmares, sleep talking, sleepwalking, limb movements or restless legs symptoms; ⑯ nocturnal nasal congestion/upper airway obstruction symptoms; ⑰ cognition, memory, attention, reaction time and daytime functioning; ⑱ mood, psychological symptoms and social interaction; ⑲ palpitations/shortness of breath; ⑳ overall symptom severity/composite symptom burden.

Non-symptom variable codes: A, demographics, body habitus and lifestyle; B, comorbidities and blood pressure; C, sleep-disordered breathing and hypoxemia measures; D, sleep architecture and arousal measures; E, craniofacial, upper airway anatomical, positional or REM-related features.

Keenan (7), and Yi (11), were international multicenter studies involving the United States, Australia, Germany, Brazil, Taiwan (China) and Iceland.

AHI, apnea–hypopnea index; BMI, body mass index; BNSQ, Basic Nordic Sleep Questionnaire; CGI-S, Clinical Global Impression–Severity; CPAP, continuous positive airway pressure; CPSS, Chinese Perceived Stress Scale; CRP, C-reactive protein; DISE, drug-induced sleep endoscopy; EDS, excessive daytime sleepiness; ESADA, European Sleep Apnea Database; ESS, Epworth Sleepiness Scale; FAMD, factor analysis of mixed data; GAD-7, Generalized Anxiety Disorder-7; HCHS/SOL, Hispanic Community Health Study/Study of Latinos; HOMA-IR, homeostatic model assessment of insulin resistance; HSAT, home sleep apnea testing; ISAC, Icelandic Sleep Apnea Cohort; ISI, Insomnia Severity Index; KoGES, Korean Genome and Epidemiology Study; LCA, latent class analysis; LPA, latent profile analysis; MAD, mandibular advancement device; MCA, multiple correspondence analysis; MD, multidiseased; MMSE, Mini-Mental State Examination; MoCA, Montreal Cognitive Assessment; ODI, oxygen desaturation index; OSA, obstructive sleep apnea; OSAS, obstructive sleep apnea syndrome; PAM, partitioning around medoids; PAP, positive airway pressure; PG, respiratory polygraphy; PHQ-9, Patient Health Questionnaire-9; PRAQ, Patient-Reported Apnea Questionnaire; PSG, polysomnography; PSQI, Pittsburgh Sleep Quality Index; PVT, psychomotor vigilance task; RDI, respiratory disturbance index; REI, respiratory event index; REI₃%, respiratory event index using a 3% oxygen desaturation criterion; REM, rapid eye movement; SAGIC, Sleep Apnea Global Interdisciplinary Consortium; SantOSA, Santiago Obstructive Sleep Apnea cohort; SAS, Self-Rating Anxiety Scale; SDS, Self-Rating Depression Scale; SF-12, 12-Item Short Form Health Survey; SHHS, Sleep Heart Health Study; SSRS, Social Support Rating Scale.

### 3.2 Phenotype-defining variables and identification methods

Symptom phenotypes were primarily identified using measures of daytime sleepiness and functioning, nocturnal sleep disturbances, respiratory interruption–related symptoms, and psychological and functional manifestations. Some studies of multidimensional clinical phenotypes also incorporated demographic characteristics, anthropometric measures, comorbidities, and objective sleep-study measures. Thirty-four study reports used standardized scales, symptom items, or composite scores (5–12, 15–18, 27–48). The Epworth Sleepiness Scale (ESS) was the most widely used instrument, appearing in 30 reports (5–12, 15–18, 27–40, 42, 43, 46–48). Insomnia and sleep quality were primarily assessed using relevant symptom items, the Insomnia Severity Index (ISI), and the Pittsburgh Sleep Quality Index (PSQI) (31, 35, 41, 44). The phenotype-defining variables and assessment tools are detailed in Table 1.

Latent variable models and cluster analysis were the predominant approaches to phenotype identification. Nineteen study reports used LCA (5–12, 15, 16, 27–30, 32, 33, 38, 39, 42), and one used latent profile analysis (LPA) (41). Other approaches included hierarchical clustering (31, 36, 37, 40, 43), k-means clustering (34, 35, 48), two-step clustering (44, 45, 47), and partitioning around medoids (19). Some studies applied dimensionality reduction before clustering (34, 44). Three additional reports used empirical or rule-based classifications based on the primary presenting complaint or combinations of ESS scores and insomnia symptoms (17, 18, 20). Substantial heterogeneity was observed across studies in phenotype-defining variables, assessment tools, analytical methods, and class labels.

### 3.3 Recurrent symptom phenotypes across studies

Based on the predominant symptom domains of the original classes and the prespecified cross-study mapping rules, three core phenotypes and two extended phenotypes were synthesized (Supplementary Table S2). The daytime sleepiness–predominant phenotype was identified in 26 study reports representing 19 independent study populations (6–12, 15–18, 20, 27–29, 31, 32, 34–36, 38–40, 42, 46, 48). Its main features were elevated ESS scores, daytime drowsiness, or an irresistible urge to sleep. The disturbed sleep/insomnia–predominant phenotype was identified in 26 study reports representing 21 independent study populations (5–12, 17, 18, 20, 27–29, 31–35, 37, 39, 41–44, 46). It was characterized primarily by difficulty initiating or maintaining sleep, frequent nocturnal awakenings, and nonrestorative sleep. The minimally symptomatic phenotype was identified in 29 study reports representing 22 independent study populations (5–12, 16–18, 20, 27–35, 37–42, 45, 48). It was characterized by low subjective symptom burden, which did not necessarily correspond to a lower respiratory event frequency, hypoxic burden, or comorbidity burden.

The upper airway/respiratory interruption symptom–predominant phenotype was identified in six study reports representing five independent study populations (7, 11, 32, 36, 43, 47). Its principal features were snoring, witnessed apneas, and nocturnal gasping or choking. The mixed-symptom/high symptom burden phenotype was identified in 15 study reports representing 13 independent study populations (5, 7, 17–19, 33, 35–38, 40, 43, 45–47). It was characterized by the simultaneous prominence of at least two symptom domains. The latter two phenotypes were classified as extended phenotypes because their original labels, symptom combinations, and phenotype-defining variables were less consistent across studies. Representative labels and detailed symptom and functional characteristics of each phenotype are presented in Table 2.

**Table 2:** Cross-study synthesis of symptom-related clinical phenotypes in adults with obstructive sleep apnea.

Synthesis level	Synthesized phenotype	Representative original class labels	Main symptom features	Reports/independent study populations
Core	Daytime sleepiness-predominant	Sleepy; excessively sleepy; sleepiness dominant; EDS phenotype	Prominent daytime sleepiness, irresistible sleepiness or dozing in specific situations, usually with elevated ESS scores; fatigue and impaired daytime functioning may coexist.	26/19
Core	Disturbed sleep/insomnia-predominant	Disturbed sleep; insomnia phenotype; COMISA; poor/non-restorative sleep	Prominent difficulty initiating or maintaining sleep, frequent nocturnal awakenings, early morning awakening or non-restorative sleep; daytime sleepiness may or may not be present.	26/21
Core	Minimally symptomatic	Minimally symptomatic; minimal symptoms; non-sleepy/non-insomnia; asymptomatic hypoxemia	Low overall burden across major symptom domains, without prominent daytime sleepiness, disturbed sleep or symptoms of respiratory interruption.	29/22
Extended	Upper airway/respiratory interruption symptom-predominant	Upper airway symptoms dominant; young snorers; sleep apnea/respiratory interruption group	Prominent snoring, witnessed apneas, nocturnal gasping or choking; sleepiness and insomnia are less prominent.	6/5
Extended	Mixed symptoms/high symptom burden	Upper airway symptoms with sleepiness; EDS-insomnia; classic symptoms; high symptom severity	High burden in at least two symptom domains, with no consistently dominant domain; specific symptom combinations vary substantially across studies.	15/13

Core and extended phenotypes are synthesized labels used in this review for descriptive cross-study synthesis. No prespecified numerical threshold for the number of contributing studies was applied, and these categories do not represent a standardized clinical classification system that has undergone formal external validation. Reports derived from the same or partially overlapping cohorts were counted as one independent study population. The disturbed sleep/insomnia-predominant phenotype represents a cross-study synthesis at the symptom level. The mixed symptoms/high symptom burden phenotype combines classes with prominent symptoms in multiple domains. The numbers of reports and independent study populations were calculated from the individual mappings and cohort grouping in Supplementary Table S2. Original labels such as less severe, moderate or severe that describe objective OSA severity do not denote levels of symptom burden. Repeated or partially overlapping samples from ISAC, SAGIC, ESADA and other cohorts were conservatively grouped.

COMISA, comorbid insomnia and obstructive sleep apnea; EDS, excessive daytime sleepiness; ESADA, European Sleep Apnea Database; ESS, Epworth Sleepiness Scale; HSAT, home sleep apnea testing; ISAC, Icelandic Sleep Apnea Cohort; OSA, obstructive sleep apnea; PSG, polysomnography; SAGIC, Sleep Apnea Global Interdisciplinary Consortium.

### 3.4 Clinical associations of symptom phenotypes

#### 3.4.1 Associations with objective disease burden

Subjective symptom burden did not show a consistent graded relationship with objective respiratory disease burden in OSA. Some minimally symptomatic or non-sleepy classes still exhibited high AHI values, substantial nocturnal hypoxemia, or a considerable comorbidity burden. Conversely, some classes characterized by disturbed sleep or high symptom burden did not have the highest AHI values or the greatest hypoxemia severity (12, 32–37, 40, 43, 45, 48). Some studies also identified classes characterized by low symptom burden with high physiological burden, or high symptom burden with relatively low physiological burden (35, 37). Symptom burden was not consistently aligned with objective measures of respiratory and hypoxemic burden, including AHI, the oxygen desaturation index (ODI), the percentage of total sleep time spent with oxygen saturation below 90% (T90), and nadir peripheral oxygen saturation (SpO_2_).

#### 3.4.2 PAP implementation and response, and patient-reported outcomes

Differences across symptom phenotypes were reported in PAP prescription and early treatment uptake. PAP prescription or early use was more common in classes with prominent sleepiness and less common in minimally symptomatic classes (9, 15, 16). No consistent differences across phenotypes were observed in mean daily PAP use or overall adherence (17–20, 46). Following PAP treatment, improvements in sleepiness and overall symptom burden were reported in most phenotypes, whereas improvements in disturbed sleep or insomnia-related symptoms were less consistent (9, 18, 19, 33). Greater sleep-related or overall symptom burden was generally associated with higher levels of anxiety, depression, and perceived stress, greater daytime functional impairment, and poorer health-related quality of life. Patients in minimally symptomatic classes reported relatively better health status (6, 12, 41, 44).

#### 3.4.3 Cardiometabolic and other long-term clinical outcomes

Minimal symptoms did not necessarily indicate low risk, and associations between symptom phenotypes and long-term cardiovascular, cerebrovascular, and metabolic outcomes were inconsistent. Some studies reported a higher prevalence of existing cardiovascular, cerebrovascular, or metabolic disease, or a higher risk of incident disease, in classes characterized by sleepiness, disturbed sleep, or high symptom burden (5, 6, 27, 38). Some longitudinal cohort studies also found that excessively sleepy or high symptom burden phenotypes were associated with increased risks of incident cardiovascular events, coronary heart disease, or heart failure (5, 27). In the EPISONO cohort, no statistically significant association was found between baseline symptom phenotype and incident cardiometabolic comorbidities (10). In a French multicenter cohort, the incidence of a composite outcome comprising major adverse cardiovascular events and all-cause mortality differed across phenotypes. However, symptom phenotypes were not independently associated with the outcome in fully adjusted models, whereas hypoxic burden and T90 remained independently associated with it (28). An elevated mortality risk was also observed among some patients with few subjective symptoms but coexisting cardiovascular disease (30). The available evidence remains insufficient to establish the independent prognostic value of symptom phenotypes for long-term clinical outcomes.

### 3.5 Longitudinal stability and transitions of symptom phenotypes

Long-term follow-up indicated that symptom phenotype membership could change over time. Over eight years of follow-up in the EPISONO cohort, approximately 45% of patients transitioned between phenotypes. The most common transition was from the minimally symptomatic to the disturbed sleep phenotype, accounting for 39% of those who transitioned. Phenotype transitions were associated with changes in AHI (10). In a five-year analysis of the Sleep Heart Health Study (SHHS) that incorporated four OSA symptom subtypes and a predefined non-OSA class, approximately 56% of participants remained in the same class, whereas approximately 44% changed class. Among the OSA symptom subtypes, the minimally symptomatic subtype showed the greatest stability and the excessively sleepy subtype the least; transitions to the moderately sleepy subtype were the most common (29). Age, sex, baseline body mass index (BMI), and baseline AHI were associated with specific transition pathways, whereas changes in AHI were not significantly associated with transitions (29). Overall, the limited longitudinal evidence suggests that symptom phenotypes can change over time, although specific transition pathways and their associated factors remain insufficiently characterized.

## 4 Discussion

### 4.1 Recurrent symptom patterns across studies of adults with OSA

This review showed that daytime sleepiness–predominant, disturbed sleep/insomnia–predominant, and minimally symptomatic patterns recurred across countries, regions, and clinical and community populations. In contrast, the upper airway/respiratory interruption symptom–predominant and mixed-symptom/high symptom burden patterns showed relatively lower consistency across studies. Notably, this recurrence was observed despite substantial heterogeneity in phenotype-defining variables, assessment tools, and identification methods, suggesting that the first three symptom patterns show relatively greater recurrence and consistency of symptom composition across study populations.

However, recurrence across studies does not constitute formal external validation. The terms “core phenotypes” and “extended phenotypes” used in this review refer to synthesized categories derived through descriptive cross-study synthesis based on recurrence across studies and consistency of symptom composition. They were not defined using prespecified quantitative thresholds and should not be regarded as an established standardized clinical classification system. Their stability and clinical applicability require further validation in independent populations.

### 4.2 Symptom phenotypes capture clinical dimensions beyond conventional respiratory event measures

This review found that symptom burden was not consistently aligned with objective respiratory disease burden. Some minimally symptomatic patients had high AHI values or substantial hypoxemia, whereas patients with disturbed sleep or high symptom burden did not necessarily have the greatest objective respiratory disease burden (12, 32–37, 40, 43, 45, 48). This discordance is consistent with the limited scope of AHI, which primarily quantifies respiratory event frequency and does not adequately capture hypoxic exposure, sleep fragmentation, sleepiness, insomnia, or impaired daytime functioning (1, 3, 4). Accordingly, when interpreting symptom-based phenotypes, ODI, T90, nadir SpO_2_, and event-related hypoxic burden should be considered as complementary measures to AHI to provide a more comprehensive assessment of the objective physiological burden of OSA (1, 3, 28, 49). The relationship between ESS scores and objective disease burden further illustrates this discordance. In a polysomnographic study of 339 participants, the median ESS score was 9 in those with moderate-to-severe OSA compared with 2 in those with normal-to-mild OSA (50). By contrast, another study reported an AHI of 32.26 events/h in patients with an ESS score of only 5.27, whereas those with an ESS score of 14.29 had an AHI of only 9.16 events/h, highlighting marked individual-level discordance between subjective sleepiness and objective respiratory burden (51).

Symptom burden, objective physiological burden, and long-term clinical risk should therefore be distinguished as three related but distinct dimensions. Minimal symptoms should not be interpreted as evidence of low physiological disease burden and should not be equated directly with low long-term risk. Similarly, high symptom burden primarily reflects greater patient-perceived and functional impacts and does not necessarily indicate the greatest hypoxic burden or cardiovascular risk. Symptom phenotypes therefore add information about the clinical expression of OSA and its impact on patients but cannot replace assessment of objective respiratory and hypoxic burden.

### 4.3 Associations of symptom phenotypes with treatment and prognosis

Some studies reported higher rates of PAP prescription or early use among patients with prominent sleepiness and lower rates among minimally symptomatic patients (9, 15, 16). Improvements in sleepiness and overall symptoms following PAP treatment were reported in most phenotypes, whereas improvements in disturbed sleep or insomnia symptoms were less consistent (9, 18, 19, 33). Associations of greater symptom burden with anxiety, depression, impaired daytime functioning, and poorer quality of life were more commonly reported (6, 12, 41, 44).

In contrast, associations of symptom phenotypes with long-term PAP adherence and cardiovascular, cerebrovascular, and metabolic outcomes were inconsistent (10, 17–20, 27, 28, 46). Although some studies linked sleepy or high symptom burden phenotypes to an increased risk of cardiovascular events, others found no independent associations. Objective measures such as hypoxic burden may offer more consistent prognostic information (27, 28). Current evidence therefore supports the clinical relevance and patient-centered value of symptom phenotypes, but does not establish them as validated tools for treatment selection or independent prognostic prediction.

### 4.4 Limited longitudinal evidence suggests that symptom phenotypes may change over time

Long-term follow-up in the EPISONO and SHHS cohorts documented transitions in symptom phenotype membership (10, 29), indicating that baseline phenotypes should not be assumed to remain stable throughout life. Symptom changes may reflect the combined influences of treatment, aging, body weight, and changes in OSA itself. The inconsistent associations between changes in AHI and phenotype transitions across the two cohorts further suggest that respiratory event frequency alone cannot explain symptom trajectories. Symptom phenotypes may therefore be better understood as descriptions of a patient's clinical state at a particular time than as lifelong labels. Their clinical relevance lies not in repeated reclassification itself, but in reassessing patients’ principal concerns when treatment, symptoms, or risk status changes and adjusting management priorities accordingly.

### 4.5 Potential implications of symptom phenotypes for multidimensional clinical assessment and long-term follow-up in OSA

The findings of this review suggest that symptom phenotypes alone cannot adequately characterize OSA severity or long-term risk. They may therefore be better considered as one component of a multidimensional clinical profile of OSA, rather than a basis for fixed patient classifications and management protocols. Precision medicine approaches to OSA have increasingly moved beyond AHI-centered assessment toward multidimensional assessment frameworks that integrate symptoms, physiological burden, comorbidities, and longitudinal monitoring data (49, 52, 53). Symptom phenotypes may thus contribute a complementary dimension reflecting clinical presentation and functional impact, alongside objective disease burden and other risk information, providing patient-centered complementary information for identifying clinical risks and needs and for informing long-term follow-up.

Within this framework, symptom phenotypes can represent the “clinical presentation and patient impact” dimension of a multidimensional OSA profile, capturing patient experiences and functional burden that conventional physiological measures do not adequately reflect. Specifically, clinical assessment may integrate symptoms and patient-reported outcomes, objective physiological burden, comorbidities and functional risk, and treatment and follow-up data to develop a more comprehensive patient profile (Figure 2).

**Figure 2:**
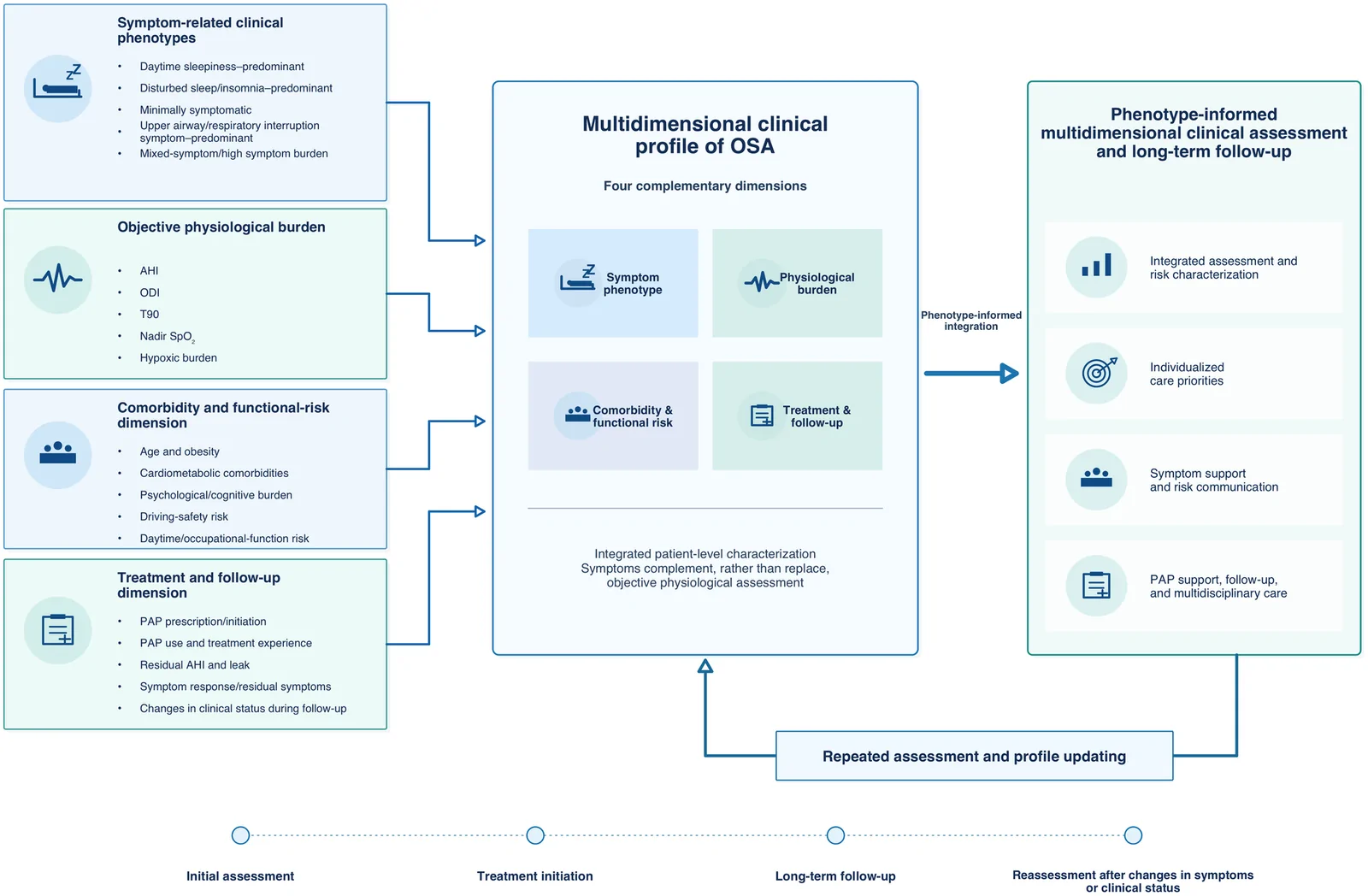
Conceptual framework for multidimensional clinical assessment and long-term follow-up incorporating symptom phenotype information in obstructive sleep apnea. The framework integrates symptom phenotypes with objective physiological burden, comorbidity and functional risk, and treatment and follow-up information to provide a more comprehensive clinical profile of OSA. It represents a conceptual synthesis of the available evidence rather than a prospectively validated phenotype-guided clinical management pathway.

Such integration may be particularly useful for interpreting the discordance between subjective symptom burden and objective physiological burden identified in this review. In patients with few symptoms but substantial objective disease burden, low subjective symptom burden should not lead to underestimation of disease burden or clinical risk. Assessment should also consider hypoxic burden, comorbidities, and the overall risk profile. Conversely, patients with high symptom burden but relatively limited respiratory burden on conventional measures warrant further evaluation for persistent insomnia, psychological distress, other sleep problems, and impaired daytime functioning. Symptom phenotypes are not intended to determine treatment decisions in isolation; rather, they may help identify the predominant current symptoms and functional burden and provide complementary information for individualized assessment and management.

Limited longitudinal evidence suggests that symptom phenotypes may change over time, indicating that a temporal dimension may also be considered in multidimensional clinical profiles of OSA. Repeated assessment of symptoms, objective physiological burden, comorbidities, and PAP use may be undertaken at initial evaluation, treatment initiation, and during long-term follow-up, with reassessment prompted by residual sleepiness, changes in insomnia, changes in body weight or comorbidities, or declining PAP use. Future research could further explore the feasibility of incorporating multidimensional assessment, identification of risks and needs, and repeated assessment into a long-term follow-up framework. Nurses can contribute to standardized symptom assessment, identification of daytime functional impairment and safety risks, monitoring of PAP data, assessment of residual symptoms, and long-term follow-up. Clinical decisions should nevertheless be based on the integration of multidimensional information and multidisciplinary judgment.

It should be emphasized that evidence linking symptom phenotypes to long-term outcomes, treatment response, and longitudinal transitions remains limited. Accordingly, the framework outlined above should be regarded as a potential approach to multidimensional clinical assessment and long-term follow-up, rather than a validated phenotype-guided clinical management pathway. Before clinical implementation, prospective studies should establish standardized and reproducible methods for individual-level symptom phenotype assignment, evaluate their stability across time and independent populations, and determine whether symptom phenotype information provides incremental clinical value beyond established physiological and clinical measures. Further clinical impact studies are needed to assess whether incorporating symptom phenotype information into multidimensional assessment and follow-up improves clinical decision-making and patient-centered outcomes compared with conventional care.

### 4.6 Strengths and limitations

Beyond synthesizing the original study classes, this review identified reports derived from the same or partially overlapping cohorts and assessed phenotype recurrence across independent study populations. It also integrated evidence on objective disease burden, treatment, patient-reported outcomes, and longitudinal transitions. Several limitations should be considered. First, the included studies differed substantially in sample sources, phenotype-defining variables, and identification algorithms. The five synthesized phenotypes represent overarching cross-study categories, rather than classes newly identified from a harmonized individual-level dataset. Second, 33 of the 36 included studies had samples in which men accounted for more than 50% of participants, and most studies recruited participants from sleep centers, resulting in some imbalance in both sex distribution and sample source. Given that women with OSA may differ in symptom presentation and clinical phenotypes, this predominantly male evidence base may limit the generalizability of the synthesized phenotypes to women with OSA and to broader community populations. Third, evidence on treatment, long-term outcomes, and phenotype transitions remains largely derived from a small number of observational cohorts. This evidence is insufficient to establish the predictive performance of symptom phenotypes or demonstrate that phenotype-guided management is superior to conventional approaches. Future research should use standardized variables, external validation across multiple centers, and longitudinal designs to further evaluate their clinical utility.

## 5 Conclusions

Recurrent symptom phenotypes have been identified across independent populations of adults with OSA, with the daytime sleepiness–predominant, disturbed sleep/insomnia–predominant, and minimally symptomatic phenotypes showing greater recurrence across studies. Symptom phenotypes capture patient-reported symptoms and functional burden that conventional physiological severity measures, such as AHI, do not adequately characterize. However, their relationships with objective disease burden, treatment response, and long-term clinical outcomes are not consistently established. Symptom phenotypes may therefore serve as a complement to objective physiological assessment and be interpreted alongside hypoxic burden, comorbidities, treatment information, and patient-reported outcomes as part of multidimensional clinical assessment, while also providing complementary information for assessing changes in symptoms, functional status, and other relevant clinical features during long-term follow-up. Future multicenter longitudinal studies should establish a standardized core set of symptom variables, strengthen external validation across populations, and further evaluate associations of symptom phenotypes with treatment response and long-term outcomes, as well as their incremental clinical value within multidimensional assessment and follow-up.

## Data Availability

The original contributions presented in the study are included in the article/Supplementary Material, further inquiries can be directed to the corresponding author.
