# Supplementary material for: Symptom-related clinical phenotypes in adults with obstructive sleep apnea: a scoping review
Source: Front Med (Lausanne). 2026 Oct 29;13:1999168. doi: 10.3389/fmed.2026.1999168 (PMC13755192; doi:10.3389/fmed.2026.1999168)
Supplement: Supplementary file 1 [file Table1.docx]

Supplementary Material

# 1 Supplementary Tables

| database | search strategy |
| --- | --- |
| pubmed: | ("sleep apnea, obstructive"[MeSH Terms] OR "obstructive sleep apnea"[Title/Abstract] OR "obstructive sleep apnoea"[Title/Abstract] OR "obstructive sleep apnea syndrome"[Title/Abstract] OR "obstructive sleep apnoea syndrome"[Title/Abstract] OR "obstructive sleep apnea-hypopnea syndrome"[Title/Abstract] OR "obstructive sleep apnea-hypopnea syndrome"[Title/Abstract] OR "obstructive sleep apnoea-hypopnoea syndrome"[Title/Abstract] OR "obstructive sleep apnoea-hypopnoea syndrome"[Title/Abstract] OR "sleep apnea-hypopnea syndrome"[Title/Abstract] OR "sleep apnea-hypopnea syndrome"[Title/Abstract] OR "sleep apnoea-hypopnoea syndrome"[Title/Abstract] OR "sleep apnoea-hypopnoea syndrome"[Title/Abstract] OR "OSA"[Title/Abstract] OR "OSAS"[Title/Abstract] OR "OSAHS"[Title/Abstract]) AND ("symptom phenotype"[Title/Abstract] OR "symptom phenotypes"[Title/Abstract] OR "symptom profile"[Title/Abstract] OR "symptom profiles"[Title/Abstract] OR "symptom pattern"[Title/Abstract] OR "symptom patterns"[Title/Abstract] OR "symptom cluster"[Title/Abstract] OR "symptom clusters"[Title/Abstract] OR "symptom subtype"[Title/Abstract] OR "symptom subtypes"[Title/Abstract] OR "clinical phenotype"[Title/Abstract] OR "clinical phenotypes"[Title/Abstract] OR "clinical subtype"[Title/Abstract] OR "clinical subtypes"[Title/Abstract] OR "cluster analysis"[Title/Abstract] OR "clustering"[Title/Abstract] OR "latent class analysis"[Title/Abstract] OR "latent profile analysis"[Title/Abstract] OR "Machine Learning"[MeSH Terms] OR "Machine Learning"[Title/Abstract] OR "unsupervised learning"[Title/Abstract] OR "data mining"[Title/Abstract]) |
| Cochrane Library： | #1 ("obstructive sleep apnea" OR "obstructive sleep apnoea" OR "obstructive sleep apnea syndrome" OR "obstructive sleep apnoea syndrome" OR "obstructive sleep apnea hypopnea syndrome") 7249  #2 ("obstructive sleep apnea-hypopnea syndrome" OR "obstructive sleep apnoea hypopnoea syndrome" OR "obstructive sleep apnoea-hypopnoea syndrome" OR "sleep apnea hypopnea syndrome" OR "sleep apnea-hypopnea syndrome") 441  #3 ("sleep apnoea hypopnoea syndrome" OR "sleep apnoea-hypopnoea syndrome" OR "OSA" OR "OSAS" OR "OSAHS") 6240  #4 ("Obstructive Sleep Apnea") 7249  #5 #1 OR #2 OR #3 OR #4 8476  #6 ("symptom phenotype" OR "symptom phenotypes" OR "symptom-related phenotype" OR "symptom-related phenotypes" OR "symptom profile") 244  #7 ("symptom profiles" OR "symptom pattern" OR "symptom patterns" OR "symptom cluster" OR "symptom clusters") 961  #8 ("symptom subtype" OR "symptom subtypes" OR "clinical phenotype" OR "clinical phenotypes" OR "clinical subtype") 522  #9 ("clinical subtypes" OR "cluster analysis" OR "clustering" OR "latent class analysis" OR "latent profile analysis") 11280  #10 ("Machine Learning" OR "machine learning" OR "unsupervised learning" OR "data mining") 4610  #11 #7 OR #8 OR #9 OR #10 OR #6 17103  #12 #5 AND #11 87 |
| web of science： | 1: TS=("symptom phenotype*" OR "symptom-related phenotype*" OR "symptom profile*" OR "symptom pattern*" OR "symptom cluster*" OR "symptom subtype*" OR ("clinical phenotype*" NEAR/5 (symptom* OR sleepiness OR insomnia OR fatigue OR depression OR anxiety OR cognition OR "quality of life" OR snoring)) OR ("clinical subtype*" NEAR/5 (symptom* OR sleepiness OR insomnia OR fatigue OR depression OR anxiety OR cognition OR "quality of life" OR snoring)) OR ("cluster analysis" NEAR/5 (symptom* OR phenotype* OR subtype* OR profile*)) OR (clustering NEAR/5 (symptom* OR phenotype* OR subtype* OR profile*)) OR ("latent class analysis" NEAR/5 (symptom* OR phenotype* OR subtype* OR profile*)) OR ("latent profile analysis" NEAR/5 (symptom* OR phenotype* OR subtype* OR profile*)) OR ("machine learning" NEAR/5 (symptom* OR phenotype* OR subtype* OR cluster* OR profile*)) OR ("unsupervised learning" NEAR/5 (symptom* OR phenotype* OR subtype* OR cluster* OR profile*)) OR ("data mining" NEAR/5 (symptom* OR phenotype* OR subtype* OR cluster* OR profile*))) Results: 59299  2: TS=("obstructive sleep apnea" OR "obstructive sleep apnoea" OR "obstructive sleep apnea syndrome" OR "obstructive sleep apnoea syndrome" OR "obstructive sleep apnea hypopnea syndrome" OR "obstructive sleep apnea-hypopnea syndrome" OR "obstructive sleep apnoea hypopnoea syndrome" OR "obstructive sleep apnoea-hypopnoea syndrome" OR "sleep apnea hypopnea syndrome" OR "sleep apnea-hypopnea syndrome" OR "sleep apnoea hypopnoea syndrome" OR "sleep apnoea-hypopnoea syndrome" OR "OSAS" OR "OSAHS" OR "OSA") Results: 89064  3: #1 AND #2 Results: 213 |
| Embase： | #206.('obstructive sleep apnea syndrome'/exp OR 204 27 Jun 2026  'obstructive sleep apnea':ti,ab,kw OR 'obstructive sleep apnoea':ti,ab,kw OR 'obstructive sleep apnea syndrome':ti,ab,kw OR 'obstructive sleep apnoea syndrome':ti,ab,kw OR 'obstructive sleep apnea hypopnea syndrome':ti,ab,kw OR 'obstructive sleep apnea-hypopnea syndrome':ti,ab,kw OR 'obstructive sleep apnoea hypopnoea syndrome':ti,ab,kw OR 'obstructive sleep apnoea-hypopnoea syndrome':ti,ab,kw OR 'sleep apnea hypopnea syndrome':ti,ab,kw OR 'sleep apnea-hypopnea syndrome':ti,ab,kw OR 'sleep apnoea hypopnoea syndrome':ti,ab,kw OR 'sleep apnoea-hypopnoea syndrome':ti,ab,kw OR 'osas':ti,ab,kw OR 'osahs':ti,ab,kw OR (('osa' NEAR/3 sleep):ti,ab,kw))  AND  ('symptom phenotype':ti,ab,kw OR 'symptom phenotypes':ti,ab,kw OR 'symptom-related phenotype':ti,ab,kw OR 'symptom-related phenotypes':ti,ab,kw OR 'symptom profile':ti,ab,kw OR 'symptom profiles':ti,ab,kw OR 'symptom pattern':ti,ab,kw OR 'symptom patterns':ti,ab,kw OR 'symptom cluster':ti,ab,kw OR 'symptom clusters':ti,ab,kw OR 'symptom subtype':ti,ab,kw OR 'symptom subtypes':ti,ab,kw OR (('clinical phenotype' NEAR/5 (symptom OR symptoms OR sleepiness OR insomnia OR fatigue OR depression OR anxiety OR cognition OR 'quality of life' OR snoring)):ti,ab,kw) OR (('clinical phenotypes' NEAR/5 (symptom OR symptoms OR sleepiness OR insomnia OR fatigue OR depression OR anxiety OR cognition OR 'quality of life' OR snoring)):ti,ab,kw) OR (('clinical subtype' NEAR/5 (symptom OR symptoms OR sleepiness OR insomnia OR fatigue OR depression OR anxiety OR cognition OR 'quality of life' OR snoring)):ti,ab,kw) OR (('clinical subtypes' NEAR/5 (symptom OR symptoms OR sleepiness OR insomnia OR fatigue OR depression OR anxiety OR cognition OR 'quality of life' OR snoring)):ti,ab,kw) OR (('cluster analysis' NEAR/5 (symptom OR symptoms OR phenotype OR phenotypes OR subtype OR subtypes OR profile OR profiles)):ti,ab,kw) OR ((clustering NEAR/5 (symptom OR symptoms OR phenotype OR phenotypes OR subtype OR subtypes OR profile OR profiles)):ti,ab,kw) OR (('latent class analysis' NEAR/5 (symptom OR symptoms OR phenotype OR phenotypes OR subtype OR subtypes OR profile OR profiles)):ti,ab,kw) OR (('latent profile analysis' NEAR/5 (symptom OR symptoms OR phenotype OR phenotypes OR subtype OR subtypes OR profile OR profiles)):ti,ab,kw) OR (('machine learning' NEAR/5 (symptom OR symptoms OR phenotype OR phenotypes OR subtype OR subtypes OR cluster OR clusters OR profile OR profiles)):ti,ab,kw) OR (('unsupervised learning' NEAR/5 (symptom OR symptoms OR phenotype OR phenotypes OR subtype OR subtypes OR cluster OR clusters OR profile OR profiles)):ti,ab,kw) OR (('data mining' NEAR/5 (symptom OR symptoms OR phenotype OR phenotypes OR subtype OR subtypes OR cluster OR clusters OR profile OR profiles)):ti,ab,kw)) |
| CINAHL： | (("obstructive sleep apnea" OR "obstructive sleep apnoea" OR "obstructive sleep apnea syndrome" OR "obstructive sleep apnoea syndrome" OR "obstructive sleep apnea hypopnea syndrome" OR "obstructive sleep apnea-hypopnea syndrome" OR "obstructive sleep apnoea hypopnoea syndrome" OR "obstructive sleep apnoea-hypopnoea syndrome" OR "sleep apnea hypopnea syndrome" OR "sleep apnea-hypopnea syndrome" OR "sleep apnoea hypopnoea syndrome" OR "sleep apnoea-hypopnoea syndrome" OR “OSA” OR “OSAS” OR “OSAHS”))  AND  ("symptom phenotype" OR "symptom phenotypes" OR "symptom-related phenotype" OR "symptom-related phenotypes" OR "symptom profile" OR "symptom profiles" OR "symptom pattern" OR "symptom patterns" OR "symptom cluster" OR "symptom clusters" OR "symptom subtype" OR "symptom subtypes" OR "clinical phenotype" OR "clinical phenotypes" OR "clinical subtype" OR "clinical subtypes" OR "cluster analysis" OR clustering OR "latent class analysis" OR "latent profile analysis" OR "machine learning" OR "unsupervised learning" OR "data mining") |
| CNKI： | 主题 = (阻塞性睡眠呼吸暂停+阻塞性睡眠呼吸暂停综合征+阻塞性睡眠呼吸暂停低通气综合征+睡眠呼吸暂停综合征+睡眠呼吸暂停低通气综合征+睡眠呼吸暂停+OSA+OSAS+OSAHS) AND (临床表型+症状表型+症状相关表型+症状群+症状簇+症状聚类+症状亚型+临床亚型+聚类分析+潜在类别分析+潜类别分析+潜在剖面分析+机器学习) |
| Wanfang： | 主题:("阻塞性睡眠呼吸暂停" OR "阻塞性睡眠呼吸暂停综合征" OR "阻塞性睡眠呼吸暂停低通气综合征" OR "睡眠呼吸暂停综合征" OR "睡眠呼吸暂停低通气综合征" OR "睡眠呼吸暂停" OR "OSA" OR "OSAS" OR "OSAHS") AND 主题:("临床表型" OR "症状表型" OR "症状相关表型" OR "症状群" OR "症状簇" OR "症状聚类" OR "症状亚型" OR "临床亚型" OR "聚类分析" OR "潜在类别分析" OR "潜类别分析" OR "潜在剖面分析" OR "机器学习") |
| VIP： | (主题=("阻塞性睡眠呼吸暂停" OR "阻塞性睡眠呼吸暂停综合征" OR "阻塞性睡眠呼吸暂停低通气综合征" OR "睡眠呼吸暂停综合征" OR "睡眠呼吸暂停低通气综合征" OR "睡眠呼吸暂停" OR "OSA" OR "OSAS" OR "OSAHS")) AND (SU=("临床表型" OR "症状表型" OR "症状相关表型" OR "症状群" OR "症状簇" OR "症状聚类" OR "症状亚型" OR "临床亚型" OR "聚类分析" OR "潜在类别分析" OR "潜类别分析" OR "潜在剖面分析" OR "机器学习")) |
| China Biology Medicine disc (CBM) | #1 常用字段 "阻塞性睡眠呼吸暂停" OR "阻塞性睡眠呼吸暂停综合征" OR "阻塞性睡眠呼吸暂停低通气综合征" OR "睡眠呼吸暂停综合征" OR "睡眠呼吸暂停低通气综合征" OR "睡眠呼吸暂停" OR "OSA" OR "OSAS" OR "OSAHS"  #2 常用字段 "临床表型" OR "症状表型" OR "症状相关表型" OR "症状群" OR "症状簇" OR "症状聚类" OR "症状亚型" OR "临床亚型" OR "聚类分析" OR "潜在类别分析" OR "潜类别分析" OR "潜在剖面分析" OR "机器学习" AND #1 |
