# Supplementary material for: Symptom-related clinical phenotypes in adults with obstructive sleep apnea: a scoping review
Source: Front Med (Lausanne). 2026 Oct 29;13:1999168. doi: 10.3389/fmed.2026.1999168 (PMC13755192; doi:10.3389/fmed.2026.1999168)
Supplement: Supplementary file 2 [file Table2.docx]

Supplementary Material

Supplementary Table S2. Cross-study mapping of original study classes to the phenotypes synthesized in this review

| **Study** | **Cohort family** | **Original study classes → synthesized phenotypes** | **Mapping rationale** |
| --- | --- | --- | --- |
| Li, 2026 (41) | — | Minimal sleep disturbance → Minimally symptomatic Mild sleep disturbance; Moderate sleep disturbance; Severe sleep disturbance → Disturbed sleep/insomnia-predominant | Classification was based solely on sleep quality. Classes were mapped according to their relative symptom burden within the study. |
| Keenan, 2018 (7) | Sleep Apnea Global Interdisciplinary Consortium (SAGIC) | Disturbed sleep → Disturbed sleep/insomnia-predominant Minimal symptoms → Minimally symptomatic Sleepiness dominant → Daytime sleepiness-predominant Upper airway symptoms dominant → Upper airway/respiratory interruption symptom-predominant Upper airway symptoms with sleepiness → Mixed symptoms/high symptom burden | Original labels and principal symptom features correspond directly to the synthesized phenotypes. |
| Pien et al., 2018 (9) | Icelandic Sleep Apnea Cohort (ISAC) | Disturbed sleep → Disturbed sleep/insomnia-predominant Minimally symptomatic → Minimally symptomatic Sleepy → Daytime sleepiness-predominant | Original labels and principal symptom features correspond directly to the synthesized phenotypes. |
| Shi, 2026 (44) | — | Moderate OSA and moderate insomnia; Moderate OSA and severe insomnia; Severe OSA and moderate insomnia → Disturbed sleep/insomnia-predominant | All participants had comorbid insomnia. Objective apnea and hypoxemia severity were treated as modifiers. |
| Bailly, 2021 (15) | European Sleep Apnea Database (ESADA) | C1: Young, obese and sleepy → Daytime sleepiness-predominant | Other classes were primarily characterized by age, body habitus, comorbidities or sleep study findings and were not mapped to symptom phenotypes. |
| González, 2021 (6) | — | Minimally symptomatic → Minimally symptomatic Excessive sleepiness → Daytime sleepiness-predominant Disturbed sleep → Disturbed sleep/insomnia-predominant | Original labels and principal symptom features correspond directly to the synthesized phenotypes. |
| Tempaku, 2022 (10) | — | Sleepy → Daytime sleepiness-predominant Minimally symptomatic → Minimally symptomatic Disturbed sleep → Disturbed sleep/insomnia-predominant | Original labels and principal symptom features correspond directly to the synthesized phenotypes. |
| Romero-Peralta,  2022 (43) | — | Young men without comorbidities, with moderate OSA and ear, nose and throat abnormalities → Upper airway/respiratory interruption symptom-predominant Nonobese women with mood disorders → Disturbed sleep/insomnia-predominant Symptomatic middle-aged obese men with very severe OSA and comorbidities; Symptomatic obese men with cardiovascular disease and severe OSA → Mixed symptoms/high symptom burden | Only classes with an identifiable symptom pattern were mapped. Body habitus, comorbidities and objective severity were treated as modifiers. |
| Pataka, 2024 (16) | European Sleep Apnea Database (ESADA) | Cluster 3: Sleepy obese women → Daytime sleepiness-predominant Cluster 4: Women with mild OSA and a low comorbidity burden → Minimally symptomatic | Other classes were primarily characterized by age or cardiovascular comorbidities and were not mapped to symptom phenotypes. |
| Saaresranta, 2016 (17) | European Sleep Apnea Database (ESADA) | EDS (daytime+/nighttime−) → Daytime sleepiness-predominant Non-EDS/non-insomnia (daytime−/nighttime−) → Minimally symptomatic EDS/insomnia (daytime+/nighttime+) → Mixed symptoms/high symptom burden Insomnia (daytime−/nighttime+) → Disturbed sleep/insomnia-predominant | Mapping was based directly on combinations of daytime sleepiness and insomnia symptoms. |
| Fontanilles Arbones, 2024 (37) | — | Middle-aged women with few symptoms and no cardiovascular risk; Older women with few symptoms, cardiovascular disease and severe OSA → Minimally symptomatic Middle-aged women with classic symptoms and cardiovascular risk → Mixed symptoms/high symptom burden Middle-aged women with mood disorders and non-restorative sleep → Disturbed sleep/insomnia-predominant | Mapping was based on the dominant symptom pattern of each class. Cardiovascular risk and objective severity were treated as modifiers. |
| Teunissen, 2025 (45) | — | High symptom severity → Mixed symptoms/high symptom burden Low symptom severity; low-symptom class with no comorbidity → Minimally symptomatic | Classes dominated by hypertension or atrial fibrillation were not mapped to symptom phenotypes. |
| Allen, 2022 (5) | — | Excessively sleepy with disturbed sleep; Moderate sleepiness with disturbed sleep → Mixed symptoms/high symptom burden Disturbed sleep without excessive sleepiness → Disturbed sleep/insomnia-predominant Minimally symptomatic → Minimally symptomatic | Mapping depended on whether multiple symptom domains were prominent. |
| Alshumrani, 2025 (20) | — | Sleepy group → Daytime sleepiness-predominant Insomniac group → Disturbed sleep/insomnia-predominant Minimally symptomatic group → Minimally symptomatic | Mapping followed the predefined classification based on the chief complaint at initial presentation. |
| Henríquez-Beltrán,  2026 (39) | Santiago Obstructive Sleep Apnea (SantOSA) cohort | Non-sleepy → Minimally symptomatic Disturbed sleep → Disturbed sleep/insomnia-predominant Excessive daytime sleepiness → Daytime sleepiness-predominant | The non-sleepy class was mapped to the minimally symptomatic phenotype based on its low overall symptom reporting. |
| Bailly, 2016 (36) | — | The drowsy obese → Daytime sleepiness-predominant The young snorers → Upper airway/respiratory interruption symptom-predominant The young symptomatic; The MD obese symptomatic → Mixed symptoms/high symptom burden | Older obese or multimorbid obese classes without a clearly dominant symptom pattern were not mapped. |
| Kim, 2018 (8) | — | Disturbed sleep → Disturbed sleep/insomnia-predominant Minimally symptomatic → Minimally symptomatic Sleepy → Daytime sleepiness-predominant | Original labels and principal symptom features correspond directly to the synthesized phenotypes. |
| Lammintausta,  2023 (18) | European Sleep Apnea Database (ESADA) | EDS without sleep complaints → Daytime sleepiness-predominant Non-EDS, non-insomnia → Minimally symptomatic EDS-insomnia → Mixed symptoms/high symptom burden Insomnia phenotype → Disturbed sleep/insomnia-predominant | Mapping was based directly on combinations of daytime sleepiness and insomnia symptoms. |
| Ida, 2022 (40) | — | Excessive sleepiness → Daytime sleepiness-predominant Nonobese and minimally symptomatic → Minimally symptomatic Classic OSA; Milder classic OSA → Mixed symptoms/high symptom burden | The older adult and severe OSA class was primarily characterized by age and objective severity and was not mapped to a symptom phenotype. |
| Gao, 2023 (38) | — | Excessively sleepy (ES); Moderately sleepy (ModS) → Daytime sleepiness-predominant Moderately sleepy with disturbed sleep (ModSwDS) → Mixed symptoms/high symptom burden Minimally symptomatic (MinS) → Minimally symptomatic | Mapping distinguished predominant sleepiness from sleepiness accompanied by disturbed sleep. |
| Morris, 2021 (42) | Sleep Heart Health Study (SHHS) | Minimally symptomatic → Minimally symptomatic Disturbed sleep → Disturbed sleep/insomnia-predominant Moderately sleepy; Excessively sleepy → Daytime sleepiness-predominant | The two classes differing in sleepiness severity were combined into one broader phenotype. |
| Van Overstraeten,  2021 (46) | — | Poorly sleeping OSAS patients with cardiometabolic comorbidities → Disturbed sleep/insomnia-predominant Younger obese men with sleepiness at the wheel → Daytime sleepiness-predominant Sleepy obese men with very severe desaturating OSAS and cardiometabolic comorbidities → Mixed symptoms/high symptom burden | Other classes were primarily characterized by objective severity or comorbidities and were not mapped to symptom phenotypes. |
| Ye, 2014 (12) | Icelandic Sleep Apnea Cohort (ISAC) | Disturbed sleep → Disturbed sleep/insomnia-predominant Minimally symptomatic → Minimally symptomatic Excessive daytime sleepiness → Daytime sleepiness-predominant | Original labels and principal symptom features correspond directly to the synthesized phenotypes. |
| Yassen, 2022 (19) | European Sleep Apnea Database (ESADA) | Young, healthy symptomatic men; Symptomatic men with psychiatric disorders; Healthy symptomatic women → Mixed symptoms/high symptom burden | Symptoms formed only part of the multidimensional clustering. Other classes were primarily characterized by body habitus, age or comorbidities and were not mapped. |
| Yi, 2026 (11) | Sleep Apnea Global Interdisciplinary Consortium (SAGIC) | Minimally symptomatic → Minimally symptomatic Disturbed sleep → Disturbed sleep/insomnia-predominant Severely sleepy; Moderately sleepy → Daytime sleepiness-predominant Upper airway symptoms dominant → Upper airway/respiratory interruption symptom-predominant | The two classes differing in sleepiness severity were combined into one broader phenotype; the remaining classes corresponded directly to the synthesized phenotypes. |
| Guo, 2019 (32) | — | Nocturnal respiratory interruption group → Upper airway/respiratory interruption symptom-predominant Daytime sleepiness group → Daytime sleepiness-predominant Insomnia and memory impairment group → Disturbed sleep/insomnia-predominant Minimally symptomatic group → Minimally symptomatic | Mapping was based on the most prominent symptom domain in each class. |
| Xia, 2026 (35) | — | Typical sleepiness → Daytime sleepiness-predominant Asymptomatic hypoxemia → Minimally symptomatic High symptom burden with mild physiological abnormalities → Mixed symptoms/high symptom burden Symptom perception-dominant → Disturbed sleep/insomnia-predominant | Objective hypoxemia and psychological features were treated as modifiers; mapping was based on the predominant subjective symptom pattern. |
| Tang, 2016 (34) | — | Daytime sleepiness → Daytime sleepiness-predominant Nocturnal insomnia → Disturbed sleep/insomnia-predominant Minimally symptomatic → Minimally symptomatic | Original labels and principal symptom features correspond directly to the synthesized phenotypes. |
| Qiu, 2021 (33) | — | Respiratory interruptions with daytime sleepiness; Severely impaired daytime functioning → Mixed symptoms/high symptom burden Minimally symptomatic → Minimally symptomatic Comorbid insomnia → Disturbed sleep/insomnia-predominant | Classes with prominent symptoms or impairment across multiple functional domains were mapped to the mixed-symptom phenotype. |
| Zhang, 2022 (48) | — | Normal weight, non-sleepy, moderate OSA; Obese, non-sleepy, severe OSA → Minimally symptomatic Obese, sleepy, very severe OSA with craniofacial restriction → Daytime sleepiness-predominant | Body habitus, hypoxemia and craniofacial features were treated as modifiers; symptom phenotypes were mapped according to the presence or absence of sleepiness. |
| Dai, 2022 (31) | — | Disturbed sleep group → Disturbed sleep/insomnia-predominant Daytime sleepiness group → Daytime sleepiness-predominant Minimally symptomatic group → Minimally symptomatic | Original labels and principal symptom features correspond directly to the synthesized phenotypes. |
| Trzepizur, 2022 (28) | — | Minimally symptomatic → Minimally symptomatic Disturbed sleep → Disturbed sleep/insomnia-predominant Excessively sleepy; Moderately sleepy → Daytime sleepiness-predominant | The study used four previously identified symptom patterns. The two classes differing in sleepiness severity were combined into one broader phenotype. |
| Morris, 2024 (29) | Sleep Heart Health Study (SHHS) | Minimally symptomatic → Minimally symptomatic Disturbed sleep → Disturbed sleep/insomnia-predominant Moderately sleepy; Excessively sleepy → Daytime sleepiness-predominant | The same four symptom classes were identified at baseline and follow-up. The two classes differing in sleepiness severity were combined into one broader phenotype. |
| Mazzotti, 2019 (25) | Sleep Heart Health Study (SHHS) | Minimally symptomatic → Minimally symptomatic Disturbed sleep → Disturbed sleep/insomnia-predominant Moderately sleepy; Excessively sleepy → Daytime sleepiness-predominant | The four symptom patterns were consistent with previous studies. The two classes differing in sleepiness severity were combined into one broader phenotype. |
| Labarca, 2020 (30) | Santiago Obstructive Sleep Apnea (SantOSA) cohort | Asymptomatic men with cardiovascular comorbidities → Minimally symptomatic The other three symptomatic classes were not mapped to a specific symptom phenotype. | Original classes were also characterized by sex and comorbidities. Available reports described the other classes only as symptomatic, providing insufficient information to identify a dominant symptom domain; these classes were therefore left unmapped. |
| Venkatnarayan, 2023 (47) | — | Younger patients with less obesity and positional OSA → Upper airway/respiratory interruption symptom-predominant Middle-aged obese patients with prominent symptoms → Mixed symptoms/high symptom burden | The older obese class with severe OSA and a high comorbidity burden was primarily characterized by age, body habitus, comorbidities and objective severity and was not mapped to a symptom phenotype. |

1. Mapping prioritized the original authors’ labels. Where labels were unclear, the dominant symptom domain was determined from within-class symptom response probabilities, means or standardized scores, cluster characteristics and the authors’ descriptions.

2. Classes differing only in severity within the same symptom domain were combined into one broader phenotype. Classes with at least two prominent symptom domains and no single dominant domain were mapped to the mixed symptoms/high symptom burden phenotype. Classes with low overall symptom burden were mapped to the minimally symptomatic phenotype.

3. Classes primarily driven by demographics, body habitus, comorbidities, objective sleep-disordered breathing measures or anatomical features, without a clear symptom pattern, were not included in the symptom phenotype synthesis.

4. Reports from the same or partially overlapping cohorts were conservatively grouped into one cohort family for cross-study counts. A single study could contribute to multiple synthesized phenotypes.

Abbreviations: EDS, excessive daytime sleepiness; MD, multidiseased; OSA, obstructive sleep apnea; OSAS, obstructive sleep apnea syndrome.
